# To the Officers and Members of the Medical Association of Ga.

**Published:** 1866-06

**Authors:** J. T. Banks

**Affiliations:** Pres. Med. Asso. of Ga.; Griffin, Ga.


### EDITORIAL AND MISCELLANEOUS.

*To the Officers and Members of the Medical Association of Ga.:*

At the last annual meeting of our Association, held in Atlanta, in 1861, I had the honor of being chosen your presiding officer. It is needless to mention the causes which have prevented any meeting since that time; they no longer exist, but the laudable purposes which first led to the organization, and which annually collected together at the appointed times and places, so many devotees to medical science, again claim our attention with increased interest.

The ability of our profession to protect itself in its purity, advance its interest and extend the domain of its usefulness, must, in a great measure, depend upon our united action. Under this impression the Association was organized. It affords the only opportunity to enlist and combine in united methodical labor, the professional talent of our State.

It is the *expressed wish* of many of our members that the Association be called together, so that its members may, again united, labor in the fields of science. From these considerations I hereby appoint a meeting of the Medical Association of Georgia, to be held in Atlanta, on the 21st of June, 1866.

In selecting the location I have been directed entirely by the wishes of members, as made known to me; Atlanta being the only place in which members of the profession have expressed a willingness to make preparations for the meeting.

The Atlanta Medical and Surgical Journal has kindly promised to publish so much as is necessary, of the proceedings of our last meeting, to which members are respectfully referred, with the hope that such special duties as were imposed on individuals and committees, then for 1862, may be discharged with reference to the appointed meeting.

As the good results flowing from our annual meetings are in proportion to the number in attendance, it is our sincere desire that every member will sacrifice upon the altar of our profession such individual interests as would otherwise prevent their attendance; and all other members of the profession, *in good standing*, are respectfully invited to attend.

The committee of arrangements will no doubt secure, as usual, transportation for the return of members over the various railroads.

J. T. BANKS,
*Pres. Med. Asso. of Ga.*

Griffin, Ga., May 15, 1866.

